# Biological Assessment of Stevioside and Sucralose as Sucrose Substitutes for Diabetics on STZ-Induced Diabetes in Rats

**DOI:** 10.3390/molecules28030940

**Published:** 2023-01-17

**Authors:** Hassan Barakat, Khaled Al-Roug, Raya Algonaiman, Sami A. Althwab, Hani A. Alfheeaid, Raghad M. Alhomaid, Mona S. Almujaydil, Taqwa Bushnaq, Tarek A. Ebeid

**Affiliations:** 1 Department of Food Science and Human Nutrition, College of Agriculture and Veterinary Medicine, Qassim University, Buraydah 51452, Saudi Arabia; 2 Food Technology Department, Faculty of Agriculture, Benha University, Banha 13736, Egypt; 3 Department of Food Science and Nutrition, College of Science, Taif University, Taif 21944, Saudi Arabia; 4 Department of Animal Production and Breeding, College of Agriculture and Veterinary Medicine, Qassim University, Buraydah 51452, Saudi Arabia; 5 Department of Poultry Production, Faculty of Agriculture, Kafrelsheikh University, Kafr El-Sheikh 33516, Egypt

**Keywords:** biological evaluation, dietary intake, histology, blood glucose monitoring, diabetes mellitus, public health practice

## Abstract

Numerous food organizations have identified excessive calorie consumption and accompanying ailments as significant health risks associated with high sugar consumption. Administering stevioside (ST), sucralose (SU), and the two synergically (SU+ST) affected normal rats’ weight gain. In the current study, SU showed the highest undesired effect. Indeed, administering the three treatments to diabetic rats (DR) did not improve the rats’ weight gain. Although, insulin injection synergically with the treatments improved the weight gain, as recorded after three weeks. The best-improving rate was observed in the ST group. After the administration of ST and ST+SU to the DR, the blood glucose level (GL) was positively affected, with SU having no effects on reducing the GL. A considerable reduction in serum insulin (SIL) was noted in the DR+SU group. On the contrary, ST did not negatively affect the SIL, rather an improvement was recorded. In addition, giving SU did not significantly affect the ALT level in the DR or normal rats (NR). A significant improvement in total bilirubin (TBILI) was observed when insulin was injected with ST or SU in DR groups. Further, triglycerides (TG) after administering ST, SU, or ST+SU to NR had no significant difference compared to the control group (NR). Although, the three treatments markedly but not significantly lowered TG in the DR. For total cholesterol (CHO), both DR and NR had no significant effect after the three treatments. No histopathological alterations were recorded in the NR group. Diffuse and severe atrophy of the islands of Langerhans due to depletion of their cells and mild papillary hyperplasia of the pancreatic ducts were represented by a slightly folded ductal basement membrane and newly formed ductules in STZ-DR. Simultaneous atrophy and absence of the cells of islands of Langerhans besides ductal hyperplasia were evident in DR+SU. Hyperplastic ductal epithelium and atrophic Langerhans cells were seen in DR+SU+In. Degeneration and mild atrophy were observed in the islands of Langerhans structures. There was essentially no noticeable change after utilizing ST. A slight shrinkage of the Langerhans’ islets was detected in DR+ST. In DR+ST+In, no histopathological alterations in the islands of Langerhans were recorded. Congestion in the stromal blood vessels associated with degenerative and necrotic changes in the cells of the islands of Langerhans in DR+SU+ST was observed. In NR+SU, congestion of the blood vessels associated with mild atrophy in the islands of Langerhans and dilatation in stromal blood vessels was noticed. In conclusion, ST is safe, and SU should be taken cautiously, such as mixing with ST and/or taken at a very low concentration to avoid its drastic effect on the human body.

## 1. Introduction

The growing concern with health and higher incidence of obesity, metabolic syndrome, and diabetes has increased interest in low-calorie food consumption [1,2]. In 2021, almost one in two adults (20–79 years old) with diabetes were unaware of their diabetes status (44.7%, 239.7 million). The highest proportions of undiagnosed diabetes (53.6%) were found in Africa, Western Pacific (52.8%), and South-East Asia regions (51.3%), respectively. The lowest proportion of undiagnosed diabetes was observed in North America and the Caribbean (24.2%) [3]. Recent reports on the prevalence of diabetes mellitus (DM), according to the International Diabetes Federation, estimated that Saudi Arabia ranked fourth in the Middle East in the number of diabetic adults (20–79 years), representing more than 4 million of the Saudi adults’ population [4]. Indeed, with increasing consumer interest in reducing sugar intake, food products made with sweeteners rather than sugar have become more popular and depleted quickly with a high market share [5]. Many foods and drinks use non-nutritive sweeteners (NNSs) to lower calorie counts without sacrificing flavor [6]. Several low-calorie products incorporating natural and/or artificial sweeteners were introduced to meet the recommended reduction of calories [7]. NNSs have only begun to develop over the past 30 years, concurrent with the increase in obesity and type 2 diabetes, which led to an increased interest in losing weight or maintaining weight loss [8,9].

NNSs are strongly recommended for use as sugar substitutes for individuals with T2D [10] because, unlike sugar, these sweeteners theoretically do not influence blood glucose concentrations when ingested. Nonetheless, previous animal studies have reported conflicting results, and thus the authentic effect of consuming artificial sweeteners in T2D remains highly debatable. Moreover, animal studies have indicated that NNSs may have synergistic or additive effects when used with other food additives [11,12]. Therefore, commercially available NNSs are used in combination to achieve the sweetness profile of sucrose and sometimes contain other biologically active ingredients such as lactose and glucose. It could be hypothesized that commercially available NNSs might have different effects, as previously reported [13].

Stevia is a natural sweetener extracted from leaves of the plant *S. rebaudiana* Bert., that produces di-terpene glycosides, which are low-calorie sweeteners. Besides having therapeutic properties, Stevia extracts contain a high level of sweetening compounds known as steviol glycosides [14,15]. Stevia contains intensely sweet substances 250 to 350 times sweeter than sugar [16]. Steviol glycosides are designated as safe (GRAS) by the FDA. Steviol glycosides can benefit those suffering from obesity, diabetes mellitus, heart disease, and dental caries [17]. Recently, it was demonstrated that the functional similarity of steviol and stevioside (ST) with that of insulin in controlling the glucose level in cell lines has been confirmed. It was evident that insulin could mimic their properties [18,19]. Additionally, dietary ST can attenuate the pro-inflammatory response after stimulation of the innate immune response [20]. Schiffman and Rother [21] concluded that sucralose (SU) interacts with chemosensors in the alimentary tract that play a role in sweet taste sensation and hormone secretion. SU intake enhanced P-glycoprotein and two cytochromes P-450 isozymes in rats’ intestines. SU can alter the microbial composition in the gastrointestinal tract (GIT), with a relatively more significant reduction in beneficial bacteria [21,22]. Early research claimed that SU passes through the GIT unmodified. However, further analysis demonstrated that some of the ingested sweeteners are metabolized in the GIT, as indicated by numerous peaks in methanolic fecal extracts after oral SU treatment. Both human and rodent studies demonstrated that SU might alter glucose, insulin, and glucagon-like peptide 1 (GLP-1) levels. These findings indicate that SU is not a biologically inert compound [23]. Experimental rats demonstrated patchy hepatocyte degeneration, Kupffer cell hyperplasia, lymphocytic infiltration, sinusoidal dilatation, and fibrosis, confirming SU-induced hepatic injury [24].

Previous studies have also evaluated SU’s chronic consumption’s toxicological and carcinogenic effects. Interestingly, there were no changes in inflammatory biomarkers, and the rats showed no signs of toxicity [25]. Although several clinical studies have consistently suggested that both acute and chronic consumption of SU has no effects on insulin, GLP-1, glucagon, and glucose homeostasis, recent animal studies have reported contradictory results [26,27,28]. Saada et al. [29] recently investigated the effects of SU administration in normal and STZ-diabetic rats at a daily dose of 11 mg kg^−1^ BW for six weeks. Their results indicated that SU administration reduces serum glucose and has no effects on serum insulin concentrations [29].

Recently, many researchers focused on the cellular and molecular mechanisms of natural products that alleviate DM, including preclinical studies and clinical trials, which will provide new insights into the treatment of diabetic nephropathy and suggest novel ideas for new drug development, as reviewed by Hu et al. [30]. Phenolics possess anti-diabetes efficiency with low toxicity or side effects, as practically observed with chlorogenic acid. It may become a useful clinical drug in treating the complex pathogenesis typical of DM and its related complications [31]. Not only phenolic acids but also flavonoids are promising drugs for treating diabetic nephropathy [32].

On the contrary, other studies using rats have reported that SU consumption enhances glucose absorption and ultimately increases blood glucose concentrations by stimulating the apical availability of GLUT-2 [33]. Hence, the effects of SU on glucose homeostasis are still contradictory. In addition, studies directly evaluating the effects of SU administration on diabetes-related parameters are limited. On the other hand, the impact of SU on the serum lipid profile is also conflicting. The clinical trial by Baird et al. [34] reported that supplementation of the SU diet at 500 mg kg^−1^ BW/day for 13 weeks has no effects on the serum lipid profile in normal humans. In a recent study by Saada et al. [29], supplementation of SU at a dose of 11 mg kg^−1^ BW/day to diabetic rats decreased TG. It elevated total cholesterol (CHO), LDL-cholesterol (LDL-c), and HDL-cholesterol (HDL-c) [29]. The differences in the results of these previous studies may be due to different experimental designs and doses used. The effects of ingestion of SU on human subjects should be investigated further. The current work aims to assess SU, ST, or their combinations as sucrose substitutes biologically using animal model experiments by determining the blood parameters (blood glucose and insulin level), liver and kidney functions, and the effect on pancreas histopathology.

## 2. Results and Discussion

### 2.1. Glucose Level

Data in Table 1 demonstrate the glucose level of STZ-induced diabetic rats over 6 weeks treated with ST, SU, and their combination with and without insulin injection. In the current study, the diabetic rats exhibited increased food and fluid intake and decreased weight gain compared to the normal rats (data not shown). After 3 h of STZ injection (45 mg kg^−1^ BW), the blood glucose level (GL) was significantly increased in the DR groups compared to the NR group. The GL (mg dL^−1^) was measured weekly for up to six weeks using fresh blood samples from rats’ tails. Results indicated that SU does not affect reducing GL, as shown in the DR+SU group. A significant reduction in GL was remarked in DR+ST and DR+SU+ST groups. However, in all treated groups with insulin, the GL was significantly (*p* < 0.05) reduced, possibly due to the external insulin effect. Non-significant differences had been observed in normal rats administrated ST, SU, or SU+ST, and results showed that ST had an improving impact on GL. Intriguingly, it has been reported that in T1D- and T2D-induced rats, daily oral ST (0.5–25 mg kg^−1^ BW) for 15 days for 6 weeks dramatically reduced GL, and improved insulin sensitivity, glucose tolerance, pancreatic insulin content [35], and fructose-fed insulin resistance in a rat model [36]. In addition, daily oral administration of another stevia glycoside, rebaudiana A, at the dose of 50–500 mg kg^−1^ BW, significantly reduced fasting blood glucose and insulin resistance. It protected β-cells in STZ-induced diabetic rats [37,38].

On the contrary, Dyrskog et al. [18] mentioned that daily oral administration of rebaudiana A at a dose of 25 mg kg^−1^ BW for 8 weeks did not improve blood glucose, serum insulin, and glucose tolerance in spontaneously diabetic rats. Administration of SU to diabetic rats did not alter GL in the present study. These results corroborate previous studies suggesting that SU consumption may not affect glucose homeostasis, body weight gain, and energy metabolism compared to control groups consuming water [26,27,34].

### 2.2. Serum Insulin Level

**Figure 1:**
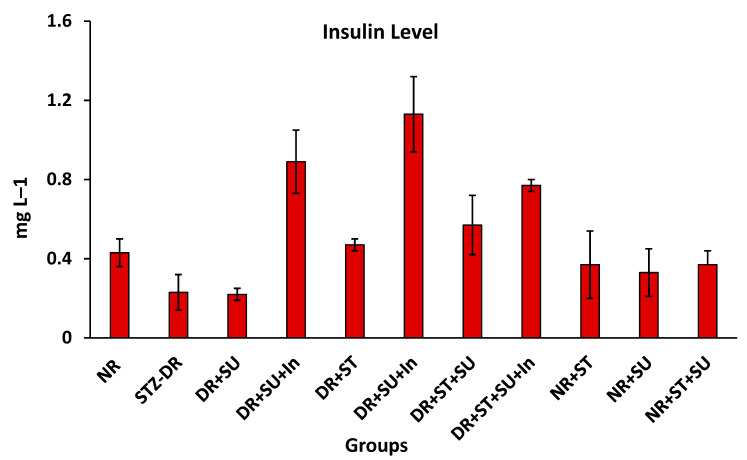
Effects of stevioside and sucralose and their combination on serum insulin in STZ-induced diabetes in rats. NR: normal rats, STZ-DR: STZ-diabetic rats, DR+SU: diabetic rats + SU, DR+SU+In: G3+insulin, DR+ST: diabetic rats+ST, DR+ST+In: G5+insulin, DR+ST+SU: diabetic rats+[SU+ST], DR+ST+SU+In: G7+insulin, NR+ST: G1+ST, NR+SU: G1+SU, NR+ST+SU: G1+[SU+ST].

Data in Figure 1 demonstrate that the serum insulin level (SIL) was affected by SU, ST, and SU+ST oral administration in the presence and absence of an external insulin injection each day with two units of insulin per dose. A significant reduction in SIL was remarked in STZ-DR as a result of β-cell dysfunction and in the DR+SU group as affected by SU administration. On the contrary, ST did not negatively affect the SIL, rather an improvement had been recorded in ST-administered groups. Additionally, groups that were given external insulin showed significant increases in SIL. However, in T2D, insulin resistance in the adipose tissue leads to defective storage of fatty acids. Thus, more fatty acids are channeled into the liver and disturb insulin signaling, ultimately increasing de novo lipogenesis by activating key transcription factors, such as SREBP1-c [39]. A slight reduction in SIL was observed in DR+SU as a non-significant effect compared to the NR group. An improvement was shown when Stevia-based sweeteners or rebaudiana A were orally taken, as mentioned by Dyrskog et al. [18] and Akbarzadeh et al. [37].

### 2.3. Liver Markers of STZ-Induced Diabetic Rats

Data in Table 2 demonstrate the two major liver function enzymes, AST and ALT, and total bilirubin of STZ-induced diabetic rats over six weeks. Diabetes caused significant increases in serum AST and ALT. Additionally, data show that the oral administration of SU did not significantly affect the ALT level in the DR or NR. Although the concentration of ALT and AST increased dramatically in the STZ-DR group compared to NR, SU did not affect the concentrations of ALT and AST in NR+SU and NR+SU+ST groups. Akbarzadesh et al. [37] reported that the daily oral administration of *S. rebaudiana* A (250, 500, and 750 mg kg^−1^ BW) did not significantly affect the AST and ALT.

On the contrary, treatment with Stevia markedly but not significantly ameliorated AST and ALT concentrations in diabetic rats [40]. Total bilirubin (TBILI) provides valuable information on the excellent functioning and performance of the liver. TBILI, a chemical breakdown product of hemoglobin, is conjugated with glucuronic acid in hepatocytes to increase its water solubility. Damaged hepatocytes may release TBILI on a large scale, leading to unconjugated hyperbilirubinemia. There was no significant effect of oral administration of SU, ST, and SU+ST on normal rats compared to the NR group given no treatment. The treatment with SU, ST, and SU+ST markedly but not significantly attenuated TBILI concentrations in diabetic rats. A significant reduction in TBILI was observed when insulin was injected with ST or SU in DR groups. These results agree with those of Abo Elnaga et al. [41] and Baird et al. [34].

### 2.4. Lipid Profile of STZ-Induced Diabetic Rats

Table 3 shows blood lipid profiles such as TG, CHO, HDL-c, LDL-c, and VLDL-c of STZ-induced diabetes in rats over six weeks. Although induction of diabetes provoked a significant increase in serum TG and CHO compared to the NR group as measured after six weeks, no significant difference was found between the NR group and NR+ST, NR+SU, and NR+SU+ST groups. Oral administration of SU, ST, and SU+ST markedly but not significantly lowered the TG in diabetic rats. No significant effect was recorded for SU, ST, and SU+ST on CHO, neither in DR nor NR groups. Akbarzadeh et al. [37] reported that daily oral administration of *S. rebaudiana* A (250, 500, and 750 mg kg^−1^ BW) significantly reduced serum TG, but no effects were observed in CHO. Dlamini [23] reported a similar finding that significantly improved serum TG, but no impact on CHO was observed.

On the contrary, Dlamini [23] indicated that SU treatment did not affect TG concentration in NR or DR groups. At the same time, SU significantly increased serum LDL-c (71%) and decreased serum HDL-c (34%) in NR, and increased CHO in DR (42%) compared to normal rates. This conflict may be due to applying different SU doses and conditions. Our results agree with Dlamini [23] in ST and show disagreement with SU results. Saada et al. [29] suggested that SU administration to diabetic rats alters the serum lipid profile. Elevated CHO in the serum of diabetic rats treated with SU may have resulted from increased absorption in the small intestine and increased de novo synthesis.

In contrast, some studies have demonstrated that SU consumption does not alter CHO in human subjects [34]. The difference in the findings might be not only due to the different experimental design and dose but also due to the different metabolic patterns of SU in other species. Administering ST alone recovered this elevation partially, to be 29.55% and 36.56% in DR+ST and DR+ST+In, respectively. The best reduction of VLDL-c was observed by administering sweeteners in DR-ST+SU+In and NR-SU+ST groups. However, slight increases in LDL-c in diabetic and normal rats might be caused by elevated lipolysis of VLDL-c, precursors for LDL-c production, by lipoprotein lipase [23]. In the same table, HDL-c, LDL-c, and VLDL-c were presented. No significant change was found in HDL-C and LDL-c after administrating SU, ST, or SU+ST. The percentage of VLDL-c was 16.8% in the NR group, and induction of diabetes significantly elevated this value to 44.73% in the STZ-DR groups. Akbarzadeh et al. [37] indicated that Stevia had no significant effect on both normal and diabetic rats.

### 2.5. Histopathological Alterations in Pancreases of STZ-Induced Diabetic Rats

**Figure 2:**
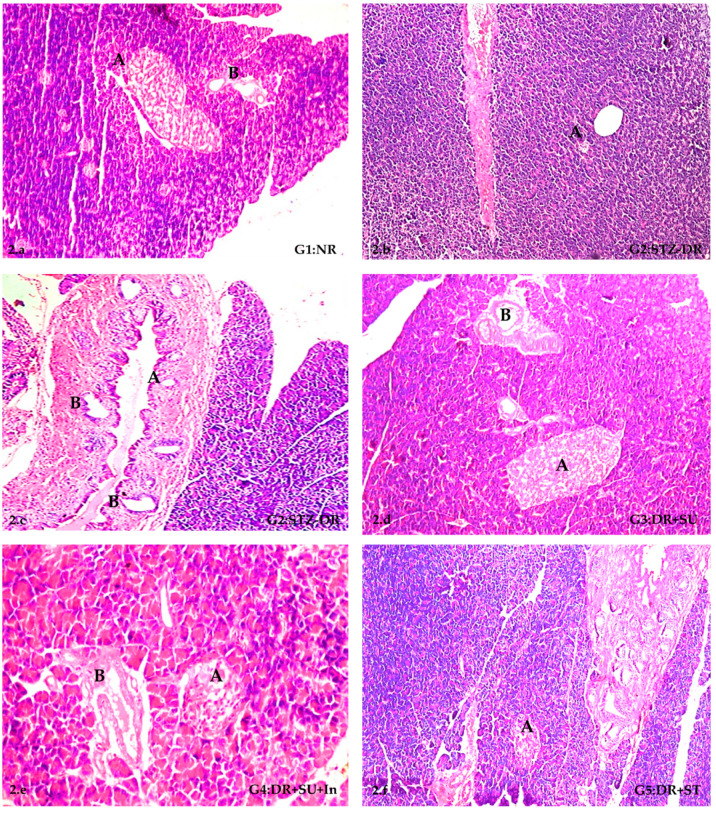

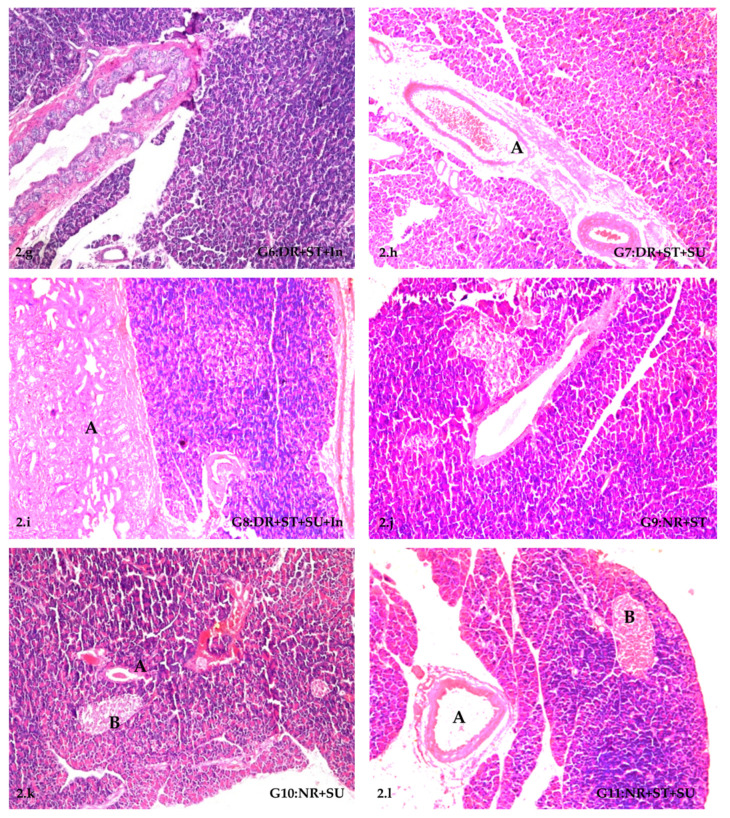
The histopathological structure in pancreatic tissues of STZ-induced diabetes in rats (H&E, X160). (**a**) Pancreas of a rat from G1 (NR) showing a typical histological structure of the islets of Langerhans (A) and the exocrine acinar and ductal system (B). (**b**) Pancreas of a rat from G2 showing complete atrophy of the islets of Langerhans (A) and (**c**) showing ductal hyperplasia (A) with newly formed ductules (B). (**d**) Pancreas of a rat from G3 showing atrophy of islets of Langerhans (A) with newly formed ductules (B). (**e**) Pancreas of a rat from G4 showing atrophy of islets of Langerhans (A) and depletion of its cells with hyperplasia of the ductal epithelium (B). (**f**) Pancreas of a rat from G5 showing mild atrophy with a degenerative change in the Langerhans cells (A). (**g**) Pancreas of a rat from G6 showing standard histological criteria. (**h**) Pancreas of a rat from G7 showing congestion in the stromal blood vessels (A). (**i**) Pancreas of a rat from G8 showing numerous newly formed ductules within a thickened stroma (A). (**j**) Pancreas of a rat from G9 showing a typical histological structure. (**k**) Pancreas of a rat from G10 showing congestion (A) and mild atrophy of pancreatic islets (B). (**l**) Pancreas of a rat from G11 showing dilatation in a stromal blood vessel (A) but the typical histological structure of the islands (B).

Initially, control rats exhibited no microscopic abnormalities in their pancreas (Figure 2a). Typical histological structures of the islets of Langerhans cells (the endocrine portion) and the acini and ducts system with the blood vessels in the stroma (exocrine one) were recorded in the NR group. Indeed, as previously mentioned, STZ was used as a diabetes inducer. Moreover, the histopathological findings of the pancreases of DR-treated rats by STZ are shown in Figure 2b–i. These findings were in the form of diffuse and severe atrophy of the islets of Langerhans due to the depletion of their cells (Figure 2b), mild papillary hyperplasia of the pancreatic ducts represented by a slightly folded ductal basement membrane, and newly formed ductules (Figure 2c). The severity of histopathological lesions was scored from (–) to (+++), as shown in Table 4. These lesions included atrophy of cells of islets of Langerhans, which was scored (+++), absence of such cells (+++), hyperplasia of the ductal epithelium (+++), and newly formed non-functioning pancreatic ductules (+++). In addition, the effect of SU as a synthetic non-nutritive sweetener with or without insulin was described in G3 (DR+SU) and G4 (DR+SU+In), respectively.

Additionally, simultaneous atrophy and absence of the cells of islets of Langerhans besides ductal hyperplasia were evident (Figure 2d). No remarkable pancreatic lesions were seen in the rats treated with SU for up to six weeks. The severity of histopathological alteration was scored as follows: atrophy of cells of islands of Langerhans (+++), absence of Langerhans cells (+++), hyperplasia of the ductal system (++), and formation of new ductules (++). In the case of G4, atrophied Langerhans cells with hyperplastic ductal epithelium were noticed (Figure 2e). A slight improvement was shown upon administration of two units of insulin injection per day for up to six weeks. Scoring of the severity of the microscopic changes was designated as (++) for atrophy of the Langerhans cells, (+) for the absence of the Langerhans cells, (++) for the papillary ductal hyperplasia, and (++) for the newly formed pancreatic ductules (Table 4). The effect of ST as a natural non-nutritive sweetener singly or in combination with insulin was described in G5 and G6, respectively. Histopathologically, the cells of islets of Langerhans showed degeneration and moderate atrophy (Figure 2f). On the other hand, no noticeable effect was observed upon the use of ST for up to six weeks. Generally, only mild atrophy of the islets of Langerhans (+) was found. In G6, there were no histopathological alterations in the islands of Langerhans (Figure 2g and Table 4).

Regarding G7, congestion was observed in the stromal blood vessels (Figure 2h) associated with degenerative and necrotic changes in the cells of the islands of Langerhans. The severity of the histopathological alterations indicated atrophy of islands of Langerhans (+++), absence of Langerhans cells (+++), hyperplasia of ductal cells (–), and newly formed ductules (–). A minor improvement was induced by the injection of two units of insulin every other day for up to six weeks in G8. However, numerous newly formed pancreatic ductules were observed (Figure 2i). Furthermore, there was thickening in the connective tissue stroma (Figure 2i), accompanied by moderate atrophy in the Langerhans cells. The severity of histopathological alterations indicated atrophy of islands of Langerhans (++), absence of Langerhans cells (–), papillary ductal hyperplasia (+), and newly formed pancreatic ductules (++). In the case of G9, no histopathological alterations were recorded (Figure 2j).

Interestingly, the normal rats administered SU alone or in combination with ST showed congestion of the blood vessels associated with mild atrophy in the islets of Langerhans (Figure 2k) and dilatation in the stromal blood vessels (Figure 2l), respectively. Oxidase results in the formation of superoxide radicals. Consequently, hydrogen peroxide and hydroxyl radicals are also generated. Furthermore, STZ liberates toxic amounts of nitric oxide that inhibit aconitase activity and participates in DNA damage. As a result of the STZ action, β-cells undergo destruction by necrosis [42].

As SU is a synthetic organochlorine sweetener, it interacts with chemosensors in the alimentary tract that play a role in sweet taste sensation and hormone secretion. SU and one of its hydrolysis products were found to be mutagenic at elevated concentrations in several testing methods. Cooking with SU at high temperatures was reported to generate chloropropanols, a potentially toxic class of compounds. Both human and rodent studies demonstrated that SU might alter glucose, insulin, and glucagon-like peptide 1 (GLP-1) levels. It is clear that STZ enters the β-cells via a glucose transporter (GLUT2) and causes the alkylation of DNA. DNA damage induces activation of poly ADP-ribosylation, a process that is more important for the diabetogenicity of STZ than DNA damage itself. Poly ADP-ribosylation leads to the depletion of cellular NAD+ and ATP. Enhanced ATP dephosphorylation after STZ treatment supplies a substrate for xanthine.

These findings indicate that SU is not a biologically inert compound [21]. Dietary administration of SU causes alterations in the histopathologic appearance of the pancreas as SU appeared to enhance the damage of the islets both in normal and diabetic rats. Additionally, hepatic degeneration appeared to be improved by the consumption of SU in both NR and DR groups [23]. Abnormalities in liver function tests are more common in type 2 diabetic patients than non-diabetic individuals and usually indicate liver tissue damage. According to Harris [43], the excess fatty acids found in the liver during an insulin-resistance state induce direct hepatocyte damage. The mechanism by which hepatocytes are damaged is not well-established. Still, several theories have been proposed, such as: disruption to the cell membrane, dysfunction of the mitochondria, formation of toxins, and disruption of key steps involved in regulating metabolism [44]. In our study, significantly increased GL concentrations and decreased insulin levels in the diabetic groups (Table 1, Figure 1) were consistent with the histopathological findings (Figure 2), which showed pancreas damage due to diabetes induction.

Research suggests that bioaccumulation of this NNS in the various tissues of both normal and diabetic rats may account for its toxicological effect. The organochlorine compound SU has been shown to bioaccumulate in animal tissues [45,46]. According to Schiffman and Rother [21], SU is an amphiphilic molecule with lipophilic and hydrophilic properties, and thus is potentially bio-accumulative. Therefore, SU or its metabolites may accumulate in the organs to cause tissue damage [23]. A recent study proved that SU has a severe effect on organs’ histopathology [24]. In addition, chronic diabetic complications, such as diabetic nephropathy, retinopathy, peripheral neuropathy, and autonomic neuropathy with the risk of cardiovascular dysfunction, might occur [23,24,47,48]. On the contrary, some studies reported Stevia extracts’ beneficial effects in improving diabetes-induced kidney damage and kidney functions in rats [49].

## 3. Materials and Methods

### 3.1. Sweeteners

ST was imported by Rebat Company for foodstuffs trade in Egypt, and SU was purchased from Bulk Supplemented Company for clean and pure bulk supplements (Lot No. 116B0806), 7511 Eastgate Road, Henderson, NV 89011.

### 3.2. Animal Model for Diabetics

Male albino Wistar rats weighing 150–200 g were housed at the Department of Food Science and Human Nutrition, College of Agriculture and Veterinary Medicine, Qassim University, Saudi Arabia, under hygienic conditions. Clean plastic cages supplied the animal rooms. The animals were allowed to be acclimatized in the laboratory environment for two weeks under a 12 h light/12 h dark cycle, with a minimum relative humidity of 40–45%, and a temperature of 23 ± 1 °C. The rats were provided tap water ad libitium. All rats received a commercial diet. All experiments and investigations were approved by the Institutional Animal Ethics Committee (IAEC) of Qassim University (Tuesday, 29 March 2022, No. 21-14-23), Saudi Arabia, implementing Regulations of the Law of Ethics of Research on Living Creatures.

### 3.3. Experimental Design

Diabetes was induced in fasted rats for 12 h by a single intraperitoneal injection of freshly prepared Streptozotocin (STZ, which is a toxic glucose analog that selectively destroys insulin-producing cells in the pancreas) (45 mg kg^−1^ body weight, dissolved in phosphate buffer (pH 4.5, Sigma Chemicals, St. Louis, MO, USA). All rats were given 5% glucose in drinking water for 24 h. Blood glucose was measured daily with a standard glucometer (Accu-CHEK, Roche, Mannheim, Germany) after collection from the tail vein in the next seven days. After 48 h of STZ treatment, rats with marked hyperglycemia (fasting blood glucose over 200 mg dL^−1^) were selected for the study and considered diabetic. Rats were then divided into eleven groups (*n* = 6), as follows: (G1): Normal control rats did not receive any treatment (NR), (G2): control diabetic rats, received STZ only (STZ-DR), (G3): diabetic rats received SU (92.5 mg kg^−1^ BW) solution daily for 6 weeks (DR+SU), (G4): diabetic rats received SU (92.5 mg kg^−1^ BW) daily + 2 units of insulin per day for 6 weeks (DR+SU+In), (G5): diabetic rats received ST (25 mg kg^−1^ BW) daily for 6 weeks (DR+ST), (G6): diabetic rats received ST (25 mg kg^−1^ BW) daily + 2 units of insulin per day for 6 weeks (DR+ST+In), (G7): diabetic rats received ST (25 mg kg^−1^ BW) + SU (92.5 mg kg^−1^ BW) for 6 weeks (DR+SU+ST), (G8): diabetic rats received ST (25 mg kg^−1^ BW) + SU (92.5 mg kg^−1^ BW) + 2 units of insulin per day for 6 weeks (DR+SU+ST+In), (G9): normal rats received ST (25 mg kg^−1^ BW) daily for 6 weeks (NR+ST), (G10): normal rats received SU (92.5 mg kg^−1^ BW) daily for 6 weeks (NR+SU), and (G11): normal rats received ST (25 mg kg^−1^ BW) + SU (92.5 mg kg^−1^ BW) for 6 weeks (NR+ST+SU). The rate doses of SU and ST were calculated as an equivalent dose of 15 mg kg^−1^ SU BW and 4 mg kg^−1^ BW for ADI in humans according to the formula provided by Reagan-Shaw et al. [50].

### 3.4. Biochemical Assessments

Two blood samples were collected from each overnight-fasted rat from the tail to follow-up on the glucose level (GL). In the end, rats were slaughtered, and blood samples were collected in dry tubes, left for 30 min at room temperature to clot, and then centrifuged at 3000 rpm for 15 min. Serums were harvested, labeled, and stored under deep-frozen conditions (−18 °C) until analysis. GL (mg dL^−1^) was determined using an enzymatic colorimetric test kit applying the GOD-PAP method. Serum was used to assess liver function parameters such as alanine aminotransferase (ALT, UL^−1^), aspartate aminotransferase (AST, UL^−1^), and total bilirubin (TBILI, mg dL^−1^) in blood serum using the alanine aminotransferase kit (EC 2.6.1.2), aspartate aminotransferase kit (EC 2.6.1.1), and photometric test kits, respectively. Lipid profiles, including TG (mg dL^−1^), CHO (mg dL^−1^), using the enzymatic colorimetric test kit applying the GPO-PAP method, and HDL-c (mg dL^−1^) using the enzymatic colorimetric direct homogenous test kit following company protocols, were determined. LDL-c (mg dL^−1^) and very low-density lipoproteins (VLDL-c, mg dL^−1^) were mathematically calculated according to Friedewald et al. [51].

### 3.5. Histological Examination

At the end of the current experiment, rats were anesthetized by diethyl ether, bled, and sacrificed. Autopsy samples were taken from the pancreas of all rats in different groups and fixed in 10% formalin saline for twenty-four hours. Washing was performed in tap water, and then serial dilutions of alcohol (methyl, ethyl, and absolute ethyl) were used for dehydration. Specimens were cleared in xylene and embedded in paraffin at 56 degrees in a hot-air oven for twenty-four hours. Paraffin bees-wax tissue blocks were prepared for sectioning at 4 microns thickness by microtome. The obtained tissue sections were collected on glass slides, deparaffinized, and stained by hematoxylin and eosin staining for routine examination through the electric light microscope [52].

### 3.6. Statistical Analysis

The statistical analysis was carried out using two-way ANOVA using SPSS, ver. 22 (IBM Corp. Released 2013). Data were treated as a complete randomization design, according to Steel et al. [53]. Multiple comparisons were carried out by applying the Tukey test, and the significance level was set at *p* < 0.05.

## 4. Conclusions

Numerous food organizations have pointed to obesity and diabetes as significant health dangers related to sugar consumption. The current work aimed to assess SU, ST, or their combinations as sucrose substitutes biologically using animal model experiments. ST is safe, and SU should be taken with caution, such as mixing with ST and/or taken at a very low concentration to avoid its drastic effects on the human body. Using ST as a sucrose replacement is safe under the ADI as it exudes no undesirable biological effects. Using SU up to the ADI showed some undesirable biological effects that may be expected when accumulated in the human body and should be monitored. Using SU+ST could be a solution to reduce the bitter aftertaste of an extra ST dose and reduce the uptake of SU. SU reduced the body weight when it was used up to its ADI in normal rats, and the results were not highly remarked in ST. SU at high doses has undesirable effects on blood parameters and organs and should be taken cautiously.

## Figures and Tables

**Table 1:** Effects of stevioside and sucralose and their combination on the glucose level (mg dL^−1^) in STZ-induced diabetes in rats, *n* = 6.

Groups	Period (Week)					
	W1	W2	W3	W4	W5	W6
NR	114.0 ^f^ ± 2.8	106.7 ^i^ ± 1.5	124.0 ^f^ ± 1.4	122.7 ^f^ ± 1.4	125.5 ^j^ ± 3.9	112.0 ^f^ ± 2.6
STZ-DR	308.7 ^ab^ ± 2.8	455.7 ^a^ ± 4.6	422.0 ^a^ ± 3.2	442.3 ^a^ ± 6.4	455.4 ^a^ ± 5.8	464.8 ^a^ ± 12.4
DR+ST	308.0 ^ab^ ± 4.9	345.0 ^c^ ± 16.0	392.7 ^b^ ± 3.9	312.7 ^cd^ ± 6.7	357.8 ^d^ ± 4.3	343.0 ^b^ ± 7.0
DR+ST+In	298.7 ^abc^ ± 5.3	296.5 ^e^ ± 1.1	281.5 ^e^ ± 23.0	282.5 ^cde^ ± 2.7	299.2 ^ef^ ± 5.0	243.5 ^de^ ± 9.3
DR+SU	298.3 ^abc^ ± 5.1	369.0 ^b^ ± 2.1	358.0 ^c^ ± 3.8	427.0 ^b^ ± 5.7	412.7 ^b^ ± 5.7	349.0 ^b^ ± 11.5
DR+SU+In	287.0 ^d^ ± 4.0	273.0 ^f^ ± 1.6	263.0 ^e^ ± 12.8	298.3 ^cd^ ± 9.8	300.8 ^ef^ ± 7.3	250.8 ^d^ ± 5.0
DR+ST+SU	318.0 ^a^ ± 3.8	329.0 ^c^ ± 9.6	327.7 ^d^ ± 4.3	314.7 ^c^ ± 7.3	378.0 ^c^ ± 3.3	337.8 ^c^ ± 6.7
DR+ST+SU+In	305.7 ^ab^ ± 4.5	309.0 ^d^ ± 9.6	307.7 ^e^ ± 4.1	284.5 ^cde^ ± 7.8	313.0 ^e^ ± 6.5	229.5 ^de^ ± 13.2
NR+ST	115.5 ^f^ ± 0.4	113.0 ^h^ ± 0.9	128.5 ^f^ ± 0.4	121.0 ^f^ ± 2.8	126.0 ^j^ ± 3.3	110.7 ^f^ ± 3.2
NR+SU	116.0 ^f^ ± 1.4	120.0 ^j^ ± 1.4	118.5 ^f^ ± 4.6	123.0 ^f^ ± 2.1	124.3 ^j^ ± 1.3	112.3 ^f^ ± 7.6
NR+ST+SU	124.0 ^e^ ± 0.7	115.0 ^h^ ± 1.4	115.0 ^f^ ± 2.1	125.5 ^f^ ± 1.1	125.3 ^j^ ± 0.8	111.3 ^f^ ± 2.0

NR: normal rats, STZ-DR: STZ-diabetic rats, DR+SU: diabetic rats + SU, DR+SU+In: G3+insulin, DR+ST: diabetic rats+ST, DR+ST+In: G5+insulin, DR+ST+SU: diabetic rats + [SU+ST], DR+ST+SU+In: G7 + insulin, NR+ST: G1 + ST, NR+SU: G1 + SU, NR+ST+SU: G1 + [SU+ST]. ^a,b,c,d,e,f,h,i,j^: No significant differences (*p* > 0.05) between any two means within the same column have the same superscripted letters.

**Table 2:** Effects of stevioside and sucralose and their combination on serum biochemical liver markers in STZ-induced diabetes in rats, *n* = 6.

Experimental Group	AST (UL^−1^)	ALT (UL^−1^)	TBILI (mg dL^−1^)
NR	194.30 ± 2.95 ^ab^	69.17 ± 2.06 ^b^	0.08 ± 0.01 ^ab^
STZ-DR	262.63 ± 14.24 ^a^	220.77 ± 72.03 ^a^	0.17 ± 0.04 ^ab^
DR+SU	183.47 ± 1.57 ^b^	127.17 ± 18.31 ^b^	0.14 ± 0.05 ^a^
DR+SU+In	177.63 ± 14.87 ^b^	81.70 ± 10.59 ^b^	0.07 ± 0.01 ^b^
DR+ST	157.27 ± 8.78 ^b^	108.03 ± 10.2 ^b^	0.10 ± 0.01 ^ab^
DR+ST+In	173.40 ± 13.89 ^a^	83.27 ± 16.99 ^b^	0.07 ± 0.04 ^b^
DR+ST+SU	167.30 ± 16.82 ^b^	99.43 ± 0.76 ^b^	0.09 ± 0.00 ^ab^
DR+ST+SU+In	166.60 ± 7.36 ^b^	82.13 ± 9.74 ^b^	0.09 ± 0.01 ^ab^
NR+ST	195.53 ± 14.78 ^ab^	58.57 ± 4.35 ^b^	0.09 ± 0.01 ^ab^
NR+SU	208.53 ± 21.03 ^ab^	68.80 ± 6.00 ^b^	0.08 ± 0.01 ^ab^
NR+ST+SU	215.23 ± 4.67 ^ab^	86.77 ± 6.83 ^b^	0.08 ± 0.01 ^ab^

^a,b^: No significant differences (*p* > 0.05) between any two means within the same column have the same superscripted letters. NR: normal rats, STZ-DR: STZ-diabetic rats, DR+SU: diabetic rats + sucralose, DR+SU+In: G3+insulin, DR+ST: diabetic rats + stevioside, DR+ST+In: G5+insulin, DR+ST+SU: diabetic rats+[SU+ST], DR+ST+SU+In: G7+insulin, NR+ST: G1+ST, NR+SU: G1+SU, NR+ST+SU: G1+[SU+ST].

**Table 3:** Effects of stevioside and sucralose and their combination on the lipid profile (mg dL^−1^) in STZ-induced diabetes in rats, *n* = 6.

Experimental Groups	TG	CHO	HDL-c	LDL-c	VLDL-c
NR	46.73 ± 3.79 ^d^	56.00 ± 3.65 ^b^	38.62 ± 2.86 ^a^	8.03 ± 0.75 ^a^	9.35 ± 0.76 ^bcd^
STZ-DR	180.30 ± 6.83 ^a^	72.93 ± 4.57 ^a^	31.99 ± 7.25 ^a^	8.87 ± 1.33 ^a^	32.08 ± 4.84 ^a^
DR+SU	160.40 ± 14.21 ^ab^	70.27 ± 4.13 ^a^	40.33 ± 3.36 ^a^	9.00 ± 0.89 ^a^	20.94 ± 5.03 ^bc^
DR+SU+In	142.33 ± 15.23 ^b^	70.33 ± 5.98 ^a^	35.47 ± 7.25 ^a^	9.80 ± 0.72 ^a^	25.79 ± 4.14 ^ab^
DR+ST	145.13 ± 19.14 ^b^	71.60 ± 10.30 ^a^	33.27 ± 1.67 ^a^	9.87 ± 0.78 ^a^	28.47 ± 10.25 ^ab^
DR+ST+In	104.70 ± 15.15 ^bc^	74.70 ± 9.92 ^a^	35.34 ± 5.94 ^a^	10.33 ± 1.69 ^a^	29.03 ± 5.83 ^ab^
DR+ST+SU	122.00 ± 6.08 ^bc^	66.57 ± 6.05 ^a^	32.17 ± 4.45 ^a^	10.00 ± 1.72 ^a^	24.40 ± 12.02 ^ab^
DR+ST+SU+In	83.17 ± 22.80 ^cd^	67.17 ± 6.50 ^a^	40.30 ± 2.05 ^a^	10.23 ± 0.95 ^a^	16.63 ± 4.56 ^bc^
NR+ST	44.93 ± 6.47 ^d^	61.87 ± 10.94 ^ab^	41.87 ± 9.90 ^a^	11.03 ± 2.14 ^a^	8.99 ± 1.29 ^bcd^
NR+SU	50.43 ± 13.29 ^d^	67.57 ± 1.58 ^a^	45.47 ± 1.94 ^a^	12.00 ± 0.46 ^a^	10.09 ± 2.66 ^bcd^
NR+ST+SU	52.73 ± 1.57 ^d^	52.67 ± 5.73 ^b^	37.50 ± 3.60 ^a^	10.00 ± 1.80 ^a^	5.15 ± 0.31 ^e^

^a,b,c,d,e^: No significant differences (*p* > 0.05) between any two means within the same column have the same superscripted letters. NR: normal rats, STZ-DR: STZ-diabetic rats, DR+SU: diabetic rats + sucralose, DR+SU+In: G3+insulin, DR+ST: diabetic rats + stevioside, DR+ST+In: G5+insulin, DR+ST+SU: diabetic rats+[SU+ST], DR+ST+SU+In: G7+insulin, NR+ST: G1+ST, NR+SU: G1+SU, NR+ST+SU: G1+[SU+ST].

**Table 4:** Scoring of the severity of the histopathological alterations in pancreatic tissues of STZ-induced diabetes in rats, *n* = 6.

Experimental Group	Atrophy of Islands of Langerhans	Absence of Langerhans Cells	Ductal Hyperplasia	Newly Formed Ductules
NR	−	−	−	−
STZ-DR	+++	+++	+++	+++
DR+SU	+++	+++	++	++
DR+SU+In	++	+	++	++
DR+ST	+	−	−	−
DR+ST+In	−	−	−	−
DR+ST+SU	+++	+++	−	−
DR+ST+SU+In	++	−	+	++
NR+ST	−	−	−	−
NR+SU	+	−	−	−
NR+ST+SU	−	−	−	−

+++ = Severe, ++ = Moderate, + = Mild, − = Nil. NR: Normal rats, STZ-DR: STZ-diabetic rats, DR+SU: diabetic rats + sucralose, DR+SU+In: G3+ insulin, DR+ST: diabetic rats + stevioside, DR+ST+In: G5+insulin, DR+ST+SU: diabetic rats + [SU+ST], DR+ST+SU+In: G7 + insulin, NR+ST: G1 + ST, NR+SU: G1+SU, NR+ST+SU: G1 + [SU+ST].

## Data Availability

Data are contained within the article.

## Sample Availability

Samples of the compounds are not available from the authors.
